# Monoallelic loss of the F-actin-binding protein radixin facilitates startle reactivity and pre-pulse inhibition in mice

**DOI:** 10.3389/fcell.2022.987691

**Published:** 2022-11-28

**Authors:** Torben J. Hausrat, Christian Vogl, Jakob Neef, Michaela Schweizer, Benjamin K. Yee, Nicola Strenzke, Matthias Kneussel

**Affiliations:** ^1^ Department of Molecular Neurogenetics, Center for Molecular Neurobiology, ZMNH, University Medical Center Hamburg-Eppendorf, Hamburg, Germany; ^2^ Institute for Auditory Neuroscience and InnerEarLab, University Medical Center Göttingen, Göttingen, Germany; ^3^ Auditory Neuroscience Group, Institute of Physiology, Medical University Innsbruck, Innsbruck, Austria; ^4^ Core Facility Morphology, Center for Molecular Neurobiology, ZMNH, University Medical Center Hamburg-Eppendorf, Hamburg, Germany; ^5^ Department of Rehabilitation Sciences, Faculty of Health and Social Sciences, and The Mental Health Research Centre, The Hong Kong Polytechnic University, Hong Kong, Hong Kong SAR, China; ^6^ Laboratory of Behavioural Neurobiology, Federal Institute of Technology Zurich, Schorenstrasse, Switzerland

**Keywords:** radixin, deafness, startle reactivity, pre-pulse inhibition, cytoskeleton, stereocilia, ERM proteins, facilitated PPI

## Abstract

Hearing impairment is one of the most common disorders with a global burden and increasing prevalence in an ever-aging population. Previous research has largely focused on peripheral sensory perception, while the brain circuits of auditory processing and integration remain poorly understood. Mutations in the *rdx* gene, encoding the F-actin binding protein radixin (Rdx), can induce hearing loss in human patients and homozygous depletion of Rdx causes deafness in mice. However, the precise physiological function of Rdx in hearing and auditory information processing is still ill-defined. Here, we investigated consequences of *rdx* monoallelic loss in the mouse. Unlike the homozygous (−/−) *rdx* knockout, which is characterized by the degeneration of actin-based stereocilia and subsequent hearing loss, our analysis of heterozygous (+/−) mutants has revealed a different phenotype. Specifically, monoallelic loss of *rdx* potentiated the startle reflex in response to acoustic stimulation of increasing intensities, suggesting a gain of function relative to wildtype littermates. The monoallelic loss of the *rdx* gene also facilitated pre-pulse inhibition of the acoustic startle reflex induced by weak auditory pre-pulse stimuli, indicating a modification to the circuit underlying sensorimotor gating of auditory input. However, the auditory brainstem response (ABR)-based hearing thresholds revealed a mild impairment in peripheral sound perception in *rdx* (+/-) mice, suggesting minor aberration of stereocilia structural integrity. Taken together, our data suggest a critical role of Rdx in the top-down processing and/or integration of auditory signals, and therefore a novel perspective to uncover further Rdx-mediated mechanisms in central auditory information processing.

## Introduction

Hearing loss refers to the partial or total inability to perceive auditory stimuli. Its global prevalence is about 20%, with over 1.5 billion people affected (Collaborators, 2021). The disability impact is likely much bigger when population aging is taken into consideration with hearing loss affecting 25% of people over the age of 60-years and almost 80% over 80-years (Oxenham, 2018). The causes of hearing loss and deafness are diverse, including genetic factors, infection, noxious noise, trauma to the ear or head and age-related sensory and neural degeneration (Stucken and Hong, 2014; Jensen et al., 2017; Bowl and Dawson, 2019; Ahmadmehrabi et al., 2021). Most of these factors affect auditory transduction in the cochlea of the inner ear (Smith et al., 2005; Oxenham, 2018; Young, 2020). However, aberrant auditory processing and/or integration in the peripheral and/or central nervous system also causes hearing loss (Powell et al., 2021; Oluwole et al., 2022; Zhang et al., 2022), but, the molecular regulation of central as opposed to peripheral auditory processing has remained far from being understood.

**FIGURE 1:**
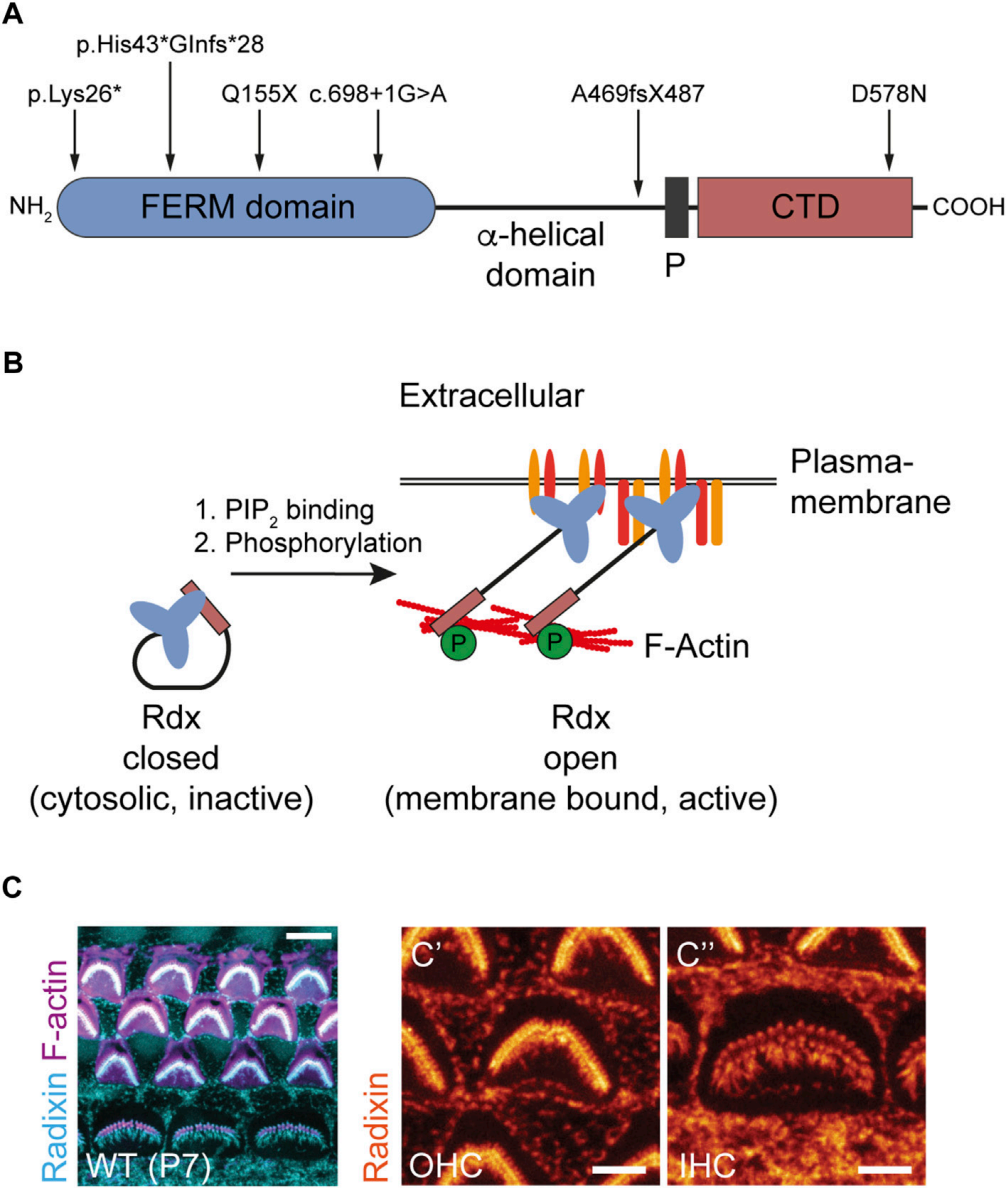
Radixin is associated with hearing loss and is expressed in the stereocilia of auditory hair cells. **(A)** Domain structure of Rdx indicating human mutations known to cause DFNB24 hearing loss. Erythrocyte Band 4.1 protein-, Ezrin-, Radixin-, Moesin-(FERM) domain harboring the PIP_2_ binding site. Proline-rich domain (P). C-terminal domain (CTD) harboring the F-actin binding site. **(B)** Schematic illustration of Rdx activation. Rdx exists in two conformational states. The closed, inactive form is mainly localized to the cytosol. Binding of its C-terminal FERM domain to PIP_2_ within the plasma membrane and subsequent phosphorylation within its C-terminal F-actin binding domain activates the protein. In its open conformation, Rdx acts as a crosslinker between the plasma membrane, integral and associated membrane proteins, and the cortical F-actin cytoskeleton. **(C)** Representative confocal microscopy maximum projection of mouse cochlear hair cell stereocilia stained for Rdx (blue) and F-actin (purple) using phalloidin-Atto647N at postnatal day 7 (P7). The magnification to the right illustrates Rdx expression (orange) in hair bundles of (C′) outer hair cells (OHCs) and (C´´) inner hair cells (IHCs) as well as microvilli at the apical surface of surrounding cells.

The ERM-family and actin-binding protein radixin are candidate molecular regulators of proper hearing. Mutations in the *rdx* gene encoding radixin (Rdx) (Figure 1A) cause non-syndromic hearing loss (DFNB24; OMIM #611022) in human patients (Khan et al., 2007; Shearer et al., 2009; Bai et al., 2019; Prasad et al., 2020). Moreover, the homozygous loss of *rdx* causes deafness in mice as result of stereocilia hair bundle degeneration (Kitajiri et al., 2004). However, a comprehensive description of Rdx function in hearing is still lacking. Together with the ERM family members ezrin and moesin, radixin shares the ability to cross-link the plasma membrane with the cortical F-actin cytoskeleton. These interactions promote the formation of a cellular scaffold pertinent to the general rigidity of cells and the mediation of F-actin-driven force that is essential for plasma membrane dynamics and the formation of filopodia, microvilli and stereocilia (Pelaseyed and Bretscher, 2018). Rdx exists in two conformational states. Its inactive cytosolic form, based on an intra-molecular interaction of its N- and the C-terminal domains, is known as the “closed” conformation. Upon binding of its N-terminal FERM-domain to PIP_2_, Rdx unfolds into an “open” conformation and relocates to the plasma membrane. Subsequently, phosphorylation of a C-terminal residue in the F-actin-binding domain stabilizes Rdx (leading to its open/active conformation), thereby linking F-actin to the plasma membrane (Figure 1B) (Hamada et al., 2000; Neisch and Fehon, 2011; McClatchey, 2014; Shabardina et al., 2016). Active Rdx is associated with different transmembrane or membrane-associated proteins, regulating their localization and function at the cell surface (Yonemura et al., 1998; Garbett and Bretscher, 2012). In the central nervous system, neuronal Rdx is an essential clustering factor of extrasynaptic GABA_A_ receptors, regulating their plasma membrane diffusion into inhibitory GABAergic synapses in an activity-dependent manner (Loebrich et al., 2006; Hausrat et al., 2015; Davenport et al., 2021).

Sensory hair cells of the inner ear are characterized by apical membrane projections, known as stereocilia. They are mechanosensing organelles essential in the transduction of sound waves into electric potentials and ultimately neural code. Stereocilia are rich in cross-linked actin filaments, which provide the scaffold for the hair-like morphology of stereocilia. Rdx is highly enriched in stereocilia of inner and outer hair cells (Figure 1C) and critically supports the transduction function of stereocilia (Kitajiri et al., 2004; Pataky et al., 2004; Prasad et al., 2020). Specifically, Rdx (in its active conformation) links up transmembrane proteins (e.g. CD44) (Kahsai et al., 2006) and scaffold proteins (e.g. NHERF2) with the submembrane actin cytoskeleton to enable effective mechanotransduction in the cochlea (Zhao et al., 2012; Shin et al., 2013; Vogl et al., 2017; Pelaseyed and Bretscher, 2018). While these data point to several critical Rdx functions in auditory sensory transduction, the question as to whether the role of Rdx extends to central auditory processing or integration has remained unexplored.

Here, we analyzed heterozygous (+/-) *rdx* knockout mice, with 50% Rdx expression relative to wildtype, and demonstrated a gain-of-function phenotype in auditory response that was in sharp contrast to the phenotypes of hearing loss and stereocilia degeneration in homozygous (−/−) *rdx* knockout mice. The gain-of-function observed in heterozygous mice included a more rapid increase of startle reactivity in response to rising acoustic stimulation and a potentiation of pre-pulse inhibition (PPI) indicative of improved detection of weak acoustic stimulation. Our data point to a so far unknown regulatory function of Rdx in central auditory processing and/or auditory-motor integration.

## Materials and methods

### PCR genotyping

Genomic DNA was isolated from tail biopsies using the Quick Extract Buffer (Biozym Scientific GmbH, Hessisch Oldendorf, Germany). For genotyping of *rdx* +/+, +/- and −/−, the following oligonucleotides were used: CAA​TTT​AAG​CCA​TGT​AGA​ATA​TCC (K10, wild-type allele); GGT​TCC​TCT​TCC​CAT​GAA​TTC (K17, knockout allele); GGA​ATT​TTG​GCA​GTA​CAT​ATT​CAG (K18). PCR product sizes: 800 base pairs for the *rdx* wild-type allele and 210 base pairs for the *rdx* knockout allele.

### Antibodies

The following primary antibodies were used: rat anti-radixin (R21, gift from S. Tsukita, WB 1:50, ICC 1:50); rabbit anti-radixin (Sigma-Aldrich, #R3653, IHC 1:200); rabbit anti-radixin (Abcam, EP 1862Y, #ab52495, IHC 1:200); mouse anti-γ-adaptin (BD Biosciences, #610386; WB 1:5,000); mouse anti-ezrin (Abcam, #ab4069, clone 3C12, WB 1:1,000); mouse anti-neuN (Millipore, clone A60, #MAB377, IHC 1:1,00). The following secondary antibodies were used: peroxidase-conjugated donkey anti-rabbit (Dianova, Hamburg, Germany, #711-036-152, WB 1:10,000); peroxidase-conjugated donkey anti-rat (Dianova, #712-036-153, WB 1:10,000); peroxidase-conjugated donkey anti-mouse (Dianova, #715-036-151, WB 1:10,000); IRDye 800CW goat anti-rabbit (LI-COR, IgG, #926-32211, WB 1:10,000); IRDye 680RD goat anti-mouse (LI-COR, IgG, #926-68070, WB 1:10,000); Alexa-488 goat anti-mouse (Dianova, #115-545-146, IHC 1:500); Cy3 donkey anti-rabbit (Dianova, #711-166-152, IHC 1:500); Cy3 donkey anti-rat (Dianova, #712-166-150, IHC 1:500); Atto488-labelled FluoTag-X4 anti-rabbit nanobody (NanoTag, IgG, #N2404, IHC 1:200). Alexa-633-coupled phalloidin (Thermo Scientific, #A22284) or Tritc-coupled phalloidin (Tebu-bio, #PHDR1) was used to visualize actin-containing stereocilia. Diamidino-2-phenylindole (DAPI, Sigma, #D9542) was used to stain the nucleus.

### Cochlea extracts

The preparation of the cochlea was described before (Vogl et al., 2017). Briefly, animals were scarified by CO_2_ exposure followed by cervical dislocation. The cochleae were rapidly removed and dissected in ice cold PBS at pH 7.4. The tissue was snap-frozen in liquid nitrogen and stored at -80°C. For total protein extraction, one cochlea per mouse was transferred into a pre-cooled 2 ml Eppendorf tube containing 500 µL ice-cold RIPA-lysis buffer (50 mM Tris-HCL pH 7.5, 1% (v/v) IGEPAL, 0.25% (w/v) Na-deoxycholat, 150 mM NaCl, 1 mM EDTA, 1 mM PMSF, 1 mM NaF (Sigma-Aldrich, St. Louis, United States), PhosSTOP phosphatases inhibitors and complete proteases inhibitors (Roche Holding AG, Basel, Switzerland)) and was incubated on ice for 1 h. To homogenize the tissue, a Teflon-plunger (Satorius AG, Göttingen, Germany) was used to crack up cochleae manually. After an additional 30-min incubation on ice, the tissue was further homogenized using a hand disperser (Polytron PT 1200 CL, Kinematic, Luzern, Switzerland) with a 3 mm aggregate and a tip speed of 4 m s^−1^ for 10 s. Afterwards, 500 µL RIPA-lysis buffer were added and the samples were kept on a rotating wheel at 4°C for additional 60 min. Subsequently, cell debris was removed by centrifugation at 1,000 x *g* for 10 min at 4°C. Supernatants were retained as total cochlea protein extracts and boiled for 6 min at 95°C in SDS-sample buffer (62.5 mM Tris-HCL pH 6.8, 10% (v/v) glycerin, 2% SDS (w/v), 5% (v/v) ß-Mercaptoethanol, 0.002% (w/v) bromphenol blue) after adjustment of protein concentrations using a BCA assay (Pierce Biotechnology, Waltham, United States). To determine protein expression levels, 10 µg protein were subjected to 10% SDS-PAGE and analyzed by western blotting.

### Western blotting

Western blot analysis was carried out as described before (Hausrat et al., 2022). Briefly, all primary antibodies were incubated in TRIS-buffered saline (TBS) supplemented with Tween-20 (TBS-T) containing 5% (w/v) dry milk (Roth, Karlsruhe, Germany) for 1 h at room temperature or, respectively, overnight at 4°C. All secondary antibodies were incubated in TBS-T containing 5% (w/v) dry milk (Roth, Karlsruhe, Germany) for 1 h at room temperature. For detection of relative immunoblot signal intensities, images were acquired using a Chemo-Cam Imager ECL HR 16-3200 (Intas) or an Odyssey CLx (LI-COR) imaging system. Signal intensities were analyzed using Fiji (ImageJ, version 2.0, NIH, United States).

### Animals and behavioral experiments

The generation of the *rdx* knockout mice has been described previously (Kikuchi et al., 2002). Male and female mice (17-week old) used in this study were single-caged under a reversed 12:12 h light/dark cycle in a temperature (22 ± 1°C) and humidity (50 ± 5%) controlled animal facility. The animals had *ad libitum* access to food and water. The behavioral experiments were conducted during the dark phase. The cohort comprised: 16 +/+ mice; 16+/- mice and 16 -/- mice. The behavioral experiments were performed at the Laboratory of Behavioural Neurobiology at the Swiss Federal Institute of Technology (ETH) Zurich and all experiments had been approved by the Zurich Cantonal Veterinary Office in compliance with the ethical standards required by the Swiss Act and Ordinance on Animal Protection, the European Council Directive 86/609/EEC, which are comparable with the National Institutes of Health Guide for Care and Use of Laboratory Animals (National Research Council (US) Institute for Laboratory Animal Research, 1996).

### Startle reactivity

The analysis of startle reactivity was previously described in (Yee et al., 2005).

#### Apparatus

The apparatus consisted of four acoustic startle chambers for mice (SR-LAB; San Diego Instruments, San Diego, CA). Each startle chamber comprised a nonrestrictive cylindrical enclosure made of clear Plexiglas attached horizontally on a mobile platform, which in turn was resting on a solid base inside a sound-attenuated isolation cubicle. A high-frequency loudspeaker mounted directly above the animal enclosure inside each cubicle produced a continuous background noise of 65 dB(A) and the various acoustic stimuli in the form of white noise with a rapid rise time of ∼1 ms. Vibrations of the Plexiglas enclosure caused by the whole-body startle response of the mouse were converted into analog signals by a piezoelectric unit attached to the platform. These signals were then digitized and stored by a computer. The sensitivity of the stabilimeter was routinely calibrated to ensure consistency between chambers and across sessions.

#### Procedures

Acoustic startle reflexes were assessed during a session lasting for approximately 30 min. The subjects were presented with a series of discrete acoustic white noise stimuli against a constant 65 dB(A) background noise. The acoustic stimuli varied randomly among 10 intensities: 69, 73, 77, 81, 85, 90, 95, 100, 110 and 120 dB(A) (which corresponded to 4, 8, 12, 16, 20, 25, 30, 35, 45 and 55 decibel units above background, respectively) and lasted either 20 or 40 ms in duration. The test began with the mice being placed in the chamber. The mice were then given a 2-min period to acclimatize to the apparatus and the continuous background noise before the first trial. The first six trials consisted of acoustic stimuli of the highest intensity only (120 dB(A), three trials with 40 ms and three with 20 ms stimulus duration) in order to stabilize the animals’ startle response. These trials were not analyzed. The animals were then presented with five blocks of discrete test trials, each comprising 20 trials, one at each stimulus intensity and stimulus duration. All trials were presented in a pseudorandom order, with a variable inter-trial interval (10 -15 s, average 13 s).

### Pre-pulse inhibition of the acoustic startle reflex

This was performed in the same apparatus as for the startle reactivity assessment, and the procedures had been described previously (Yee et al., 2005; Dubroqua et al., 2015). The same animals that undergone the startle reactivity test have been tested in PPI. Briefly, the animals were presented with a series of discrete trials, each comprising a weak acoustic stimulus (pre-pulse) that shortly followed by a startle-eliciting burst of acoustic stimulus (pulse). PPI refers to the diminution of the startle reaction to the pulse due to the preceding pre-pulse. All stimuli were presented against a constant background noise of 65 dB(A). The duration of pre-pulse and pulse stimuli were 20 and 40 ms, respectively, and always in sequence with a stimulus onset asynchrony (SOA) of 100 ms. The intensity of the pulse stimuli was set at 100, 110, or 120 dB(A). The intensity of the pre-pulse was set at: 65, 71, 77, or 83 dB(A). When the pre-pulse was set at 65 dB(A), the trials were effectively pulse-alone (or no-pre-pulse) trials, against which PPI was evaluated.

A test session began by placing the mouse into the Plexiglas holder. After a 2-min acclimatization period, the animal was presented with six pulse-alone trials (two at each pulse intensities), to habituate and stabilize the animal’s startle response. They were not included in the analysis. The animals were then presented with six blocks of discrete trials. Each block comprised 16 trials: 12 were formed by 3 levels of pulse with 4 levels (including background only) of pre-pulse, and 4 additional pre-pulse-only trials at 65 dB(A) (i.e., background), 71, 77, or 83 dB(A). The 16 discrete trials within each block were presented in a pseudorandom order, with a variable inter-trial interval of 15 s (ranging from 10 to 20 s). The total duration of the PPI test session was approximately 30 min. The whole-body startle reaction of the mouse was measured on each trial within a time window of 65 ms (from the onset of the pulse in pulse-alone and pre-pulse-*plus*-pulse trials, or the onset of the pre-pulse on pre-pulse-alone trials). This output (in arbitrary units) was referred to as the reactivity score. PPI was specifically indexed by percent inhibition (%PPI), defined as the relative reduction in startle reaction on pre-pulse-*plus*-pulse trials relative to pulse-alone trials and calculated at each pre-pulse intensity as follows: [1 − (reaction in pre-pulse-*plus*-pulse trials)/(reaction in pulse-alone trials)] × 100%.

### Stereocilia imaging

Whole cochleae were isolated from 4-month-old *rdx* (+/+), (+/-) and (−/−) littermate mice. Cochleae were immediately immersed in 4% PFA in 0.1 M phosphate buffer. Cochleae were manually perfused with this buffered fixative through the oval and round windows, post-fixed over night at room temperature (RT) and washed with phosphate buffered saline, pH 7.4 and decalcified in 10% EDTA (pH = 8) for 1 week at RT with slight agitation. For whole-mount preparations, fixed and decalcified cochleae were transferred to PBS and the otic capsule, spiral ligament, Reissner’s membrane and the tectorial membrane were removed under a dissection microscope. Half-turn segments of the organ of Corti and spiral ganglion were dissected and transferred into 6-well culture plates for staining as floating preparations. Cochlear pieces were stained for radixin using a rat primary antibody (R21; dilution 1:50) in PBS containing 1% horse serum overnight at 4°C, followed by a Alexa-488 conjugated secondary goat anti-rat antibody (dilution 1:1,000) and Phalloidin Rhodamin (dilution 1:1,000) for 1.5 h in PBS with 1% horse serum at RT. Whole mounts were then carefully removed, placed on individual SuperFrost Ultra plus™ glass slides (Menzel Glaeser) and mounted in Aqua Polymount (Polysciences Inc., Eppelheim, Germany). Images of stereocilia on outer hair cells were acquired using a laser scanning confocal microscope (Olympus FV-1000) equipped with a 63x objective, in a sequential scanning mode with equal settings across genotypes. Images were saved as overlay TIFF files for subsequent analysis using MetaMorph 7.1 (Universal Imaging, Downingtown, PA).

### Immunostainings and confocal/super-resolution (STED) microscopy of cochlear samples

Immunostaining of freshly dissected apical cochlear turns was performed as described previously (Kroll et al., 2019) with slight modifications: cochleae of postnatal day (P)7 or 17-week-old mice were fixed with 4% formaldehyde in phosphate-buffered saline (PBS)—either for 1 h (on ice) or overnight (at 4°C). Next, specimens were washed (PBS) and permeabilized (30 min in PBS +0.5% Triton-X100) and then incubated for 1 h in blocking buffer (PBS +10% goat serum +0.5% Triton-X100) in a wet chamber at RT. Primary antibodies were diluted in blocking buffer and applied for 1 h at RT or overnight at 4°C in a wet chamber. After extensive washing with PBS, the tissue was incubated with secondary antibodies and/or fluorophore-conjugated phalloidin in a light-protected wet chamber for 1 h at RT. Then, the specimens were washed in PBS and finally mounted onto glass microscope slides with Mowiol mounting medium. The following primary antibodies and phalloidin-conjugates were employed in this study: rabbit anti-radixin (1:200, R3653, Sigma Aldrich; Rdx-KO-verified in (Vogl et al., 2017)), phalloidin-Atto488 (1:200; Cat. No. 49409Merck) or phalloidin-Atto647N; (1:200; Cat. No. 65906 Merck). Phalloidins were either applied directly after permeabilization (STED) or together with secondary reagents for multi-target labelings. To visualize radixin, we used an Atto488-labelled FluoTag®-X4 anti-rabbit IgG nanobody (N2404, NanoTag). Confocal z-stacks and 2D-STED images were acquired with a pixel size of 60x60x150 nm (xyz: confocal) or 15 × 15 nm (xy: STED) on an Abberior Instruments Expert Line 775 nm 2-color STED microscope (Abberior Instruments), with excitation laser lines at 485, 561 and 640 nm and a 1.2 W emission-depletion laser at 775 nm, using a 100x/1.4 NA oil immersion objective. Images were processed using ImageJ. Stereocilia full-width at half-maximum (FWHM) was determined with custom-written code in Igor Pro 7. Final image assembly for display was prepared using Adobe Illustrator.

### Immunohistochemistry

Adult mice were scarified by CO_2_ exposure and perfused using PBS containing 1,000 U/ml heparin (Ratiopharm), following 4% PFA/PBS (w/v). Brains were post-fixed for 12 h in 4% PFA/PBS. Serial sagittal sections of 50 μm thickness were obtained using a vibratome (Leica, VT 1000S). Free-floating sections were kept in PBS at 4°C until further processing. For immunohistochemistry, sections were rinsed in 0.1 M phosphate buffer (0.06659 M sodium phosphate di-basic heptahydrate, 0.03341 M sodium phosphate mono-basic monohydrate, pH 7.2). Unspecific epitopes were blocked for 1 h in 0.1 M phosphate buffer containing 5% donkey serum (v/v), and 0.1% Triton X-100 (v/v). Primary antibodies (rabbit anti-radixin (Abcam), mouse anti-neuN (Millipore)) were incubated over night at 4°C diluted in 0.1 M phosphate buffer containing 1% donkey serum (v/v), 1% BSA (w/v) and 0.1% Triton X-100 (v/v). The following day, sections were washed three times in 0.1 M phosphate buffer for 15 min at RT and subsequently incubated with fluorescently-tagged secondary antibodies over night at 4°C in 0.1 M phosphate buffer containing 1% donkey serum (v/v), 1% BSA (w/v) and 0.1% Triton X-100 (v/v). Next, diamidino-2-phenylindole (DAPI, 0.2 μg/ml) was added and incubated for 30 min at RT. Afterwards, sections were washed four times for 15 min at RT using 0.1 M phosphate buffer. Finally, sections were rinsed in H_2_O, mounted in Aqua Poly/Mount (Polysciences, Warrington, PA, United States, #18606) and dried overnight in the dark at RT.

Images of the frontal cortex and the underlying striatum were acquired using a laser scanning confocal microscope (Olympus FV-1000) equipped with a 20x objective, in a sequential scanning mode with equal settings across genotypes. Series of three 2D images (z-stacks) with a step size of 2.26 µm of each region of interest were acquire and saved as overlay TIFF files. Images were further processed and analyzed using Fiji (ImageJ, version 2.0, NIH, United States). Images were stitched using the “pairwise stitching” plugin. Stitched images were projected using “maximal intensity projection” function and converted to a RGB format. Average signal intensities were analyzed in regions of interest (ROI) within the frontal cortex and the dorsal striatum.

### ABR and DPOAE measurements

Auditory brainstem responses (ABR) and product otoacoustic emissions (DPOAE) measurements were carried out using Tucker Davis System III hardware as described before (Jing et al., 2013). Briefly, mice were anesthetized by ketamine (125 mg/kg, i.p.) and xylazine (2.5 mg/kg, i.p.) and placed on a heat blanket (Hugo Sachs Elektronik, Harvard Apparatus). For ABR, stimuli were presented with a JBL 2402 speaker through BioSig Software (TDT) and the EEG between vertex and mastoid was averaged at least 2 × 1,300 times with a differential amplifier (Neuroamp, gain 50.000, filter 400-4000 Hz). For recording DPOAE, the ED1/EC1 speaker system (equipped with a Sennheiser MKE-2 microphone and a Terratec DMX6 Fire USB sound card) controlled by a custom MATLAB software (MathWorks) was used.

### Statistical analysis

At least 3 biologically independent repeats were conducted for each experiment. Statistical analyses were performed with either SPSS (Chicago, IL, United States) or Prism (GraphPad Software Inc., CA, United States). Briefly, after an exploratory data analysis, data were checked for normality using Kolmogorov-Smirnov or Shapiro-Wilk tests. For comparison, either two-tailed independent Student *t*-test or one-, two-, three- or four-way ANOVA were used. Graphs were constructed using Excel (Microsoft, Redmond, WA, United States) or Prism. Normally distributed data are shown as bar diagrams and individual data points are shown as dots, if *n* < 10. Statistical significance was defined as follows: *p ≤ 0.05, **p ≤ 0.01, ***p ≤ 0.001. More detailed inferential statistics for individual experiments are outlined in the respective figure legends.

## Results

### Increased startle reactivity in heterozygous *rdx* knockout mice

Autosomal recessive deafness is linked to homozygous mutations in the human *rdx* gene (Khan et al., 2007; Shearer et al., 2009; Bai et al., 2019; Prasad et al., 2020) affecting different domains of the protein (Figure 1A). In line with this, homozygous (−/−) *rdx* knockout mice suffer from a developmental degeneration of cochlear hair cell stereocilia that leads to hearing loss (Kitajiri et al., 2004). Although these phenotypes indicate an essential role of *rdx* in cochlear function, the underlying pathological mechanisms have remained elusive.

**FIGURE 2:**
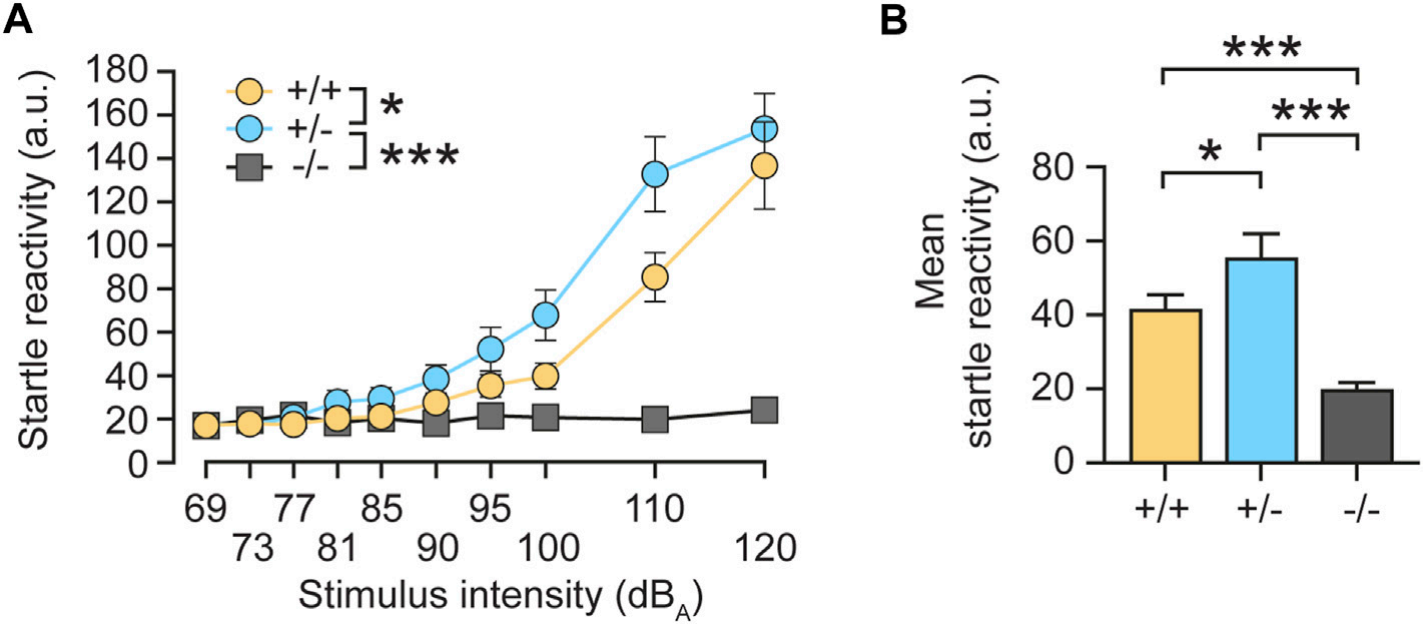
Monoallelic loss of rdx increases the acoustic startle reflex in mice. **(A)** The intensity of the startle reaction (average of 10 trials, arbitrary units (a.u.)) expressed as a function of stimulus intensity. *Rdx* knockout (−/−) mice displayed no startle reactivity during increasing stimulus intensities, as compared to wild-type (+/+) or heterozygote (+/-) animals. The startle responses of heterozygotes (+/-) revealed higher overall startle reactivity compared to wildtype (+/+) controls above 81 dB_A_. **(B)** Mean startle reactivity across all stimulus intensities shown in **(A)**. Data were obtained from *n* = 16 (+/+), 16 (+/-) and 16 (−/−) adult *rdx* knockout mice, with equal numbers of males and females for each genotype. ANOVA was used to assess statistical significance. *p < 0.05, ***p < 0.001. Data represent mean ± SEM.

To further investigate a physiological role of radixin in hearing, we aimed to study a behavioral response to auditory stimulation following monoallelic *rdx* gene deletion in heterozygous (+/-) *rdx* knockout mice. First, we assessed the acoustic startle reflex. As expected, homozygous (−/−) *rdx* knockouts almost completely eliminated the acoustic startle response (Figure 2A, grey). By contrast, heterozygous (+/-) mice exhibited a more prominent startle response (Figure 2A, blue) compared with wildtype (+/+) control animals (Figure 2A, yellow). This phenotype emerged clearly from 81 dB(A) onwards until asymptote at 120 dB(A) and was independent of sex. These findings were supported by a 3 × 2 × 10 (genotype x sex x stimulus intensity) ANOVA of the average startle reactivity scores (10 trials collapsed across stimulus duration), which yielded a significant main effect of genotype [F(2,42) = 20.60, *p* < 0.0001] and of genotype × intensity interaction [F(18,378) = 16.665, *p* < 0.0001]. The analysis also yielded a main effect of sex [F(1,42) = 4,513, *p* < 0.05] but not its interaction with the genotype [F(2,42) = 1,978, *p* > 0.15]. Consistent with our interpretation, subsequent post-hoc comparisons confirmed a significant difference between homozygous knockouts and wild-type controls (Figure 2B, *p* < 0.0001). The higher mean startle response of heterozygotes (+/-) relative to wildtype controls was also confirmed to be significant (Figure 2B; *p* < 0.02). We therefore concluded that monoallelic loss of *rdx* leads to a gain-of-function phenotype with respect to acoustic startle sensitivity. This phenotype might stem from 1) reduced Rdx protein expression that impacts the structural and/or physiological function of the cochlea and consequently affects sensory transduction of stimuli in the inner ear, and/or 2) facilitation of sensory processing and/or the integration of stimuli in the startle pathway that connects the auditory input to startle reaction.

### Monoallelic loss of radixin only slightly impairs sensory auditory function

**FIGURE 3:**
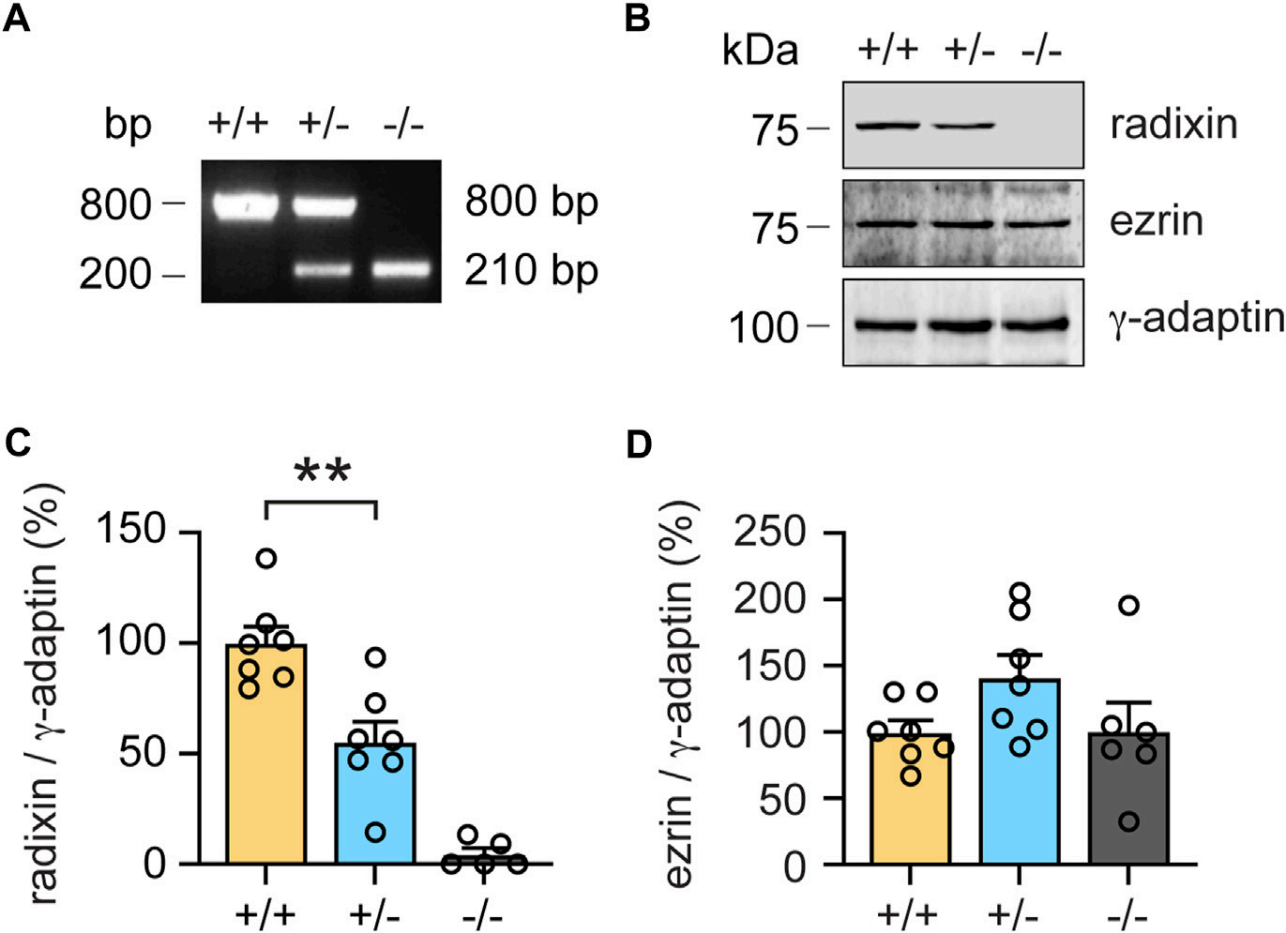
Monoallelic loss of rdx reduces radixin protein expression in the cochlea. **(A)** Representative genotyping PCR of wildtype (+/+), heterozygous (+/-) and homozygous (−/−) *rdx* knockout mice. Base pairs (bp). **(B)** Representative western blot analysis of radixin (Rdx) and ezrin (Ezr) in cochlea extracts derived from (+/+), (+/-) and (−/−) *rdx* knockout mice. *γ*-adaptin was used as loading control. Kilodalton (kDa). **(C,D)** Quantification of Rdx **(C)** and Ezr **(D)** signal intensities normalized to *γ*-adaptin as shown in **(B)**. (+/+) set to 100%. N = 7-5 mice per genotype. Independent Student´s *t*-test was used to assess statistical significance. **p < 0.01. Data represent mean ± SEM.

To investigate the first scenario, we quantified Rdx protein expression in the cochlea of heterozygous (+/-) mice. Based on PCR genotyping (Figure 3A) we also isolated cochlear extracts derived from (+/+), (+/-) and (−/−) mice, which were subsequently analyzed by western blotting. Cochleae derived from homozygous (−/−) *rdx* KO animals did not express radixin protein, whereas cochleae from heterozygous (+/-) KOs displayed a reduction of about 50% protein expression in the inner ear relative to wildtype littermates (Figures 3B,C; independent Student’s *t*-test; *p* = 0.0027). An analysis of ezrin (Ezr) expression, the closest homologue of Rdx, revealed a trend indicative of a compensatory upregulation in (+/-) and (−/−) mice, as compared to wildtype (+/+) levels (Figure 3B). However, this upregulation failed to reach statistical significance in (+/-) mice (*p* = 0.0532, independent Student’s *t*-test) and (−/−) mice (*p* = 0.982) (Figure 3D).

**FIGURE 4:**
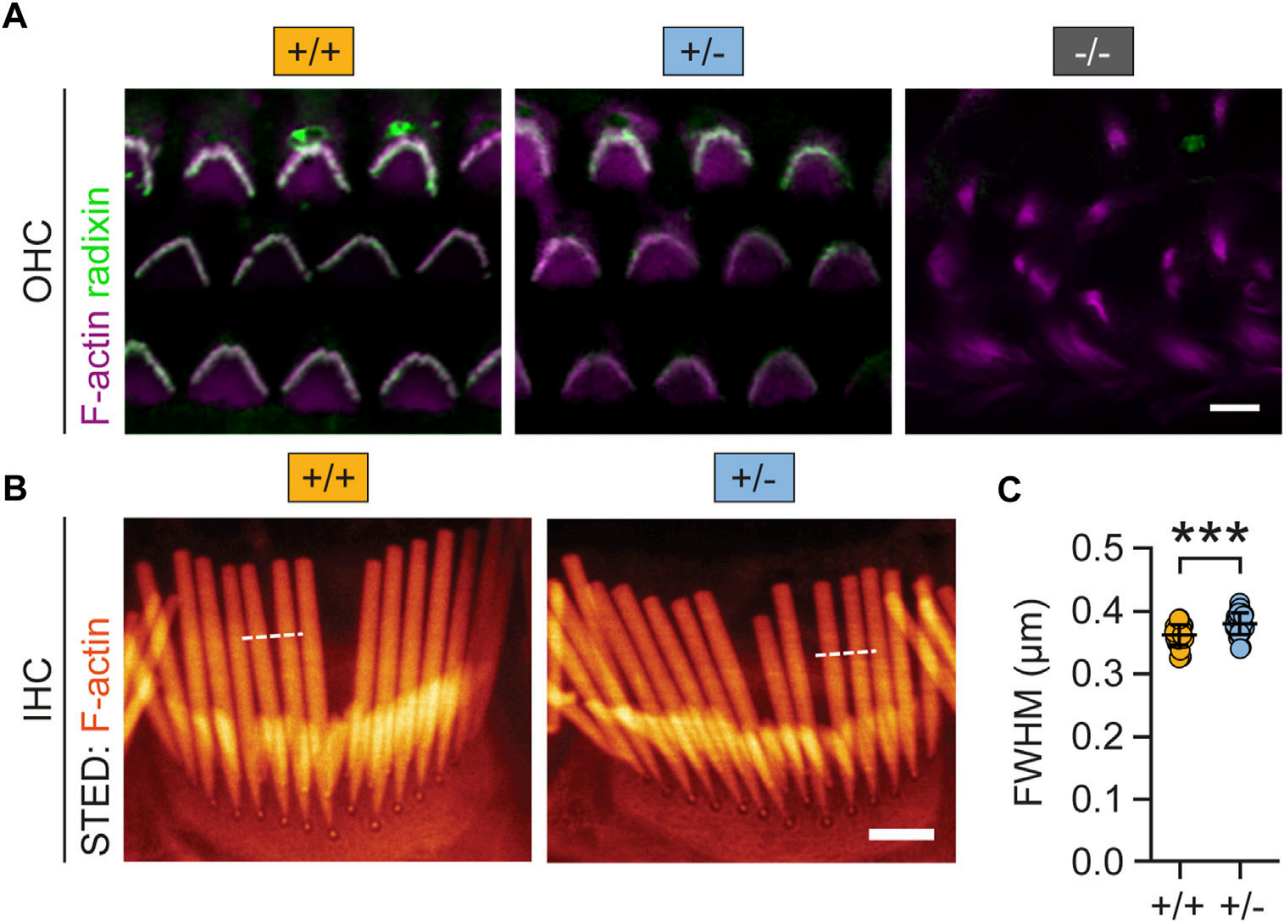
Monoallelic loss of rdx mildly alters auditory hair cell stereocilia morphology. **(A)** Representative confocal micrographs of mouse cochlear outer hair cell (OHC) stereocilia immunostained for Rdx (green) and F-actin (Tritc-Phalloidin; purple) derived from adult (+/+), (+/-) and (−/−) *rdx* knockout mice. Rdx is expressed in hair bundles of OHCs. The overall morphology of stereocilia appears comparable between (+/+) and (+/-) but it was severely degenerated in (−/−) tissue. **(B)** Representative super-resolution STED images of phalloidin-labelled (orange) inner hair cell (IHC) bundles, derived from (+/+) and (+/-) mice. **(C)** Quantification of full width at half maximum (FWHM) values derived from fluorescence intensity profiles (white dashed lines). Data revealed a small but statistically significant increase in stereociliar width in *rdx* (+/-), compared to wildtype (+/+) mice. (+/+): n(stereocilia) = 236, n(IHC) = 29, N = 3; (+/-): n(stereocilia) = 213; n(IHC) = 30, N = 3. Independent Student´s *t*-test was used to asses statistical significance. ***p < 0.001. Data represent grand averages per IHC ± SD.

Next, we tested whether reduced Rdx expression altered the morphology of auditory hair cell stereocilia. To this end, we combined fluorescent phalloidin with a Rdx-specific antibody to visualize actin-rich stereocilia and the structure of outer hair cell (OHC) bundles (Kitajiri et al., 2004). As expected, confocal microscopy revealed that the total loss of radixin (*rdx* −/− mice) leads to the degeneration of hair cell stereocilia (Figure 4A, right). In contrast, the morphology of stereocilia in heterozygous (+/-) mice was comparable with wildtype (+/+) controls (Figure 4A, left, middle). Quantitative super-resolution microscopy, using stimulated emission microscopy (STED) imaging of inner hair cells (IHC) confirmed this impression. The overall stereocilia morphology of phalloidin-labelled IHC bundles appeared indistinguishable between (+/+) and (+/-) *rdx* knockout mice (Figure 4B). However, quantification of the stereociliar full width at half maximum (FWHM) revealed a small but significant thickening in heterozygous (+/-) *rdx* knockout mice as compared with wild-type (+/+) controls (Figure 4C; independent Student’s *t*-test; *p* = 0.0001). Together, these findings suggest that a monoallelic loss of *rdx* is not detrimental for stereociliar structural integrity.

**FIGURE 5:**
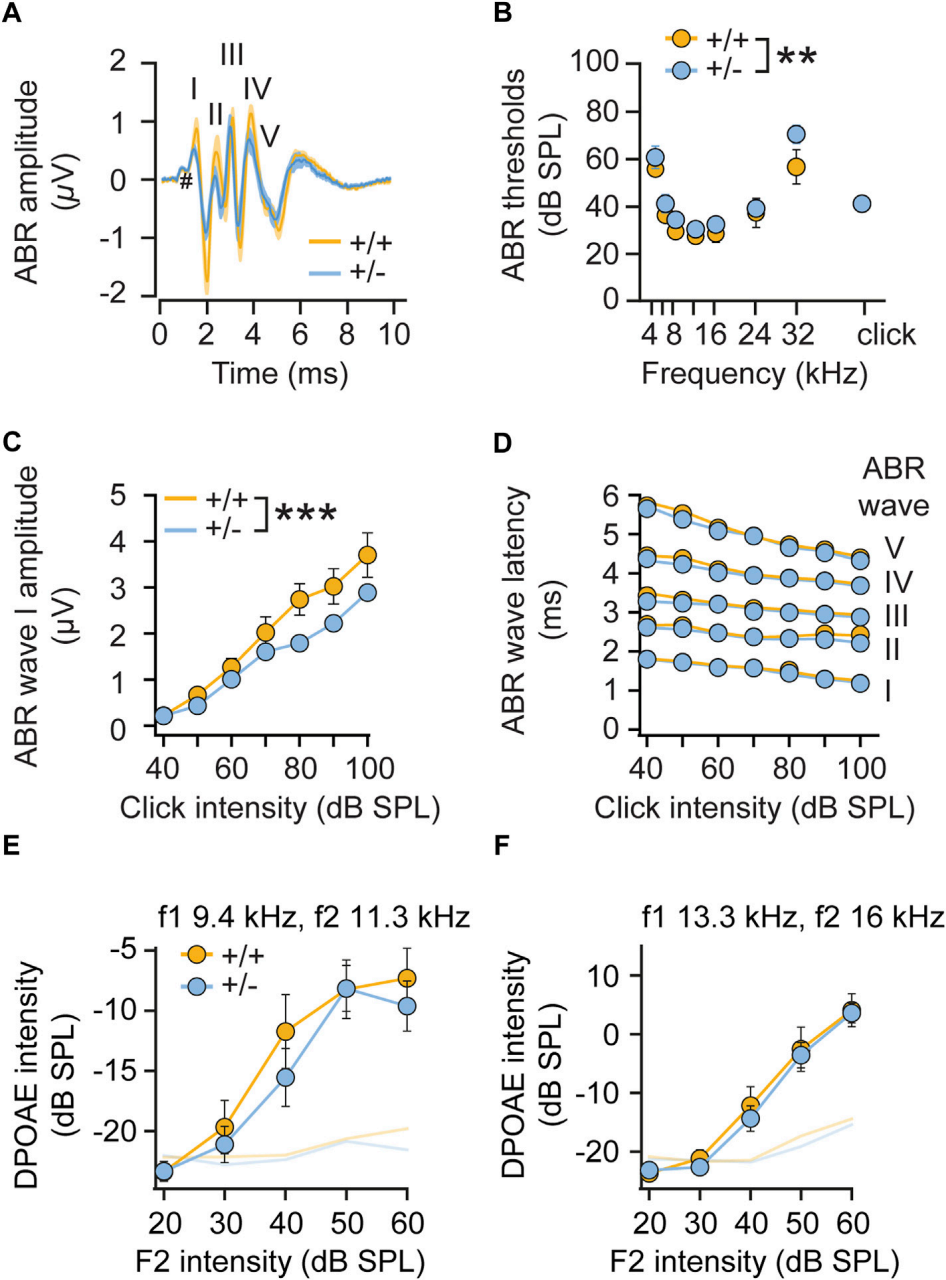
Monoallelic loss of radixin only minimally affects hearing function. **(A)** ABR waveforms to a 80-dB click stimulation in adult (+/+) and (+/-) *rdx* mice (grand averages ± SEM, *n* = 10 each) are well preserved. Roman numerals on top designate ABR waves; # indicates the summating potential. **(B)** ABR thresholds to tone burst stimulation are slightly elevated in *rdx* (+/-) mice (*p* = 0.0074, 2-way ANOVA across all tone burst frequencies; *p* = 0.08, independent Student’s t-test for clicks). **(C)** ABR wave I amplitude is slightly reduced in *rdx* (+/-) mice (*p* < 0.0001, 2-way ANOVA). **(D)** Analysis of ABR latencies show no differences across genotypes. **(E,F)** Analysis of DPOAE growth functions (shown for f1 9.4 kHz, f2 11.3 kHz **(E)** and f1 = 13.3kHz/f2 16 kHz **(F)**, L2 = L1-10) revealed no significant differences across genotypes. *n* = 10 mice per genotype. 2-way ANOVA was used to assess statistical significance. **p < 0.01, ***p < 0.001. Data represent grand averages ± SEM.

Given the elevated startle reactivity in heterozygous (+/-) *rdx* knockout mice (Figure 2), we further assessed hearing function in more detail by recording auditory brainstem responses (ABRs). ABR waveforms, reflecting synchronous action potential generation in the auditory nerve (wave I) and auditory brainstem (waves II-V), were largely comparable between genotypes (Figure 5A). We observed a minimal increase in ABR thresholds in *rdx* (+/-) mice (Figure 5B) to tone burst stimulation (*p* = 0.0074, two-way ANOVA across all tone burst frequencies; *p* = 0.08, independent *t*-test for clicks) and a slight decrease in ABR amplitude (Figures 5A,C), indicating a marginal impairment of hair cell and/or spiral ganglion neuron function. A detailed analysis of ABR waves II-V amplitudes (Figure 5A) and latencies (Figure 5D) did not reveal any major defects of signal propagation in auditory pathways. Furthermore, the analysis of amplitudes of distortion product otoacoustic emissions (DPOAE, reflecting active cochlear amplification by outer hair cells, Figures 5E,F) in heterozygous knockout mice was close to wild type levels. Overall, we observed only minimal cochlear dysfunction that was consistent with marginal deficits in hair cell transduction. However, this effect is inconsistent with the observed phenotype of increased startle reactivity. It is therefore plausible that this gain-of-function phenotype in heterozygous (+/-) *rdx* knockout mice (Figure 2) cannot be attributed to aberrant sensory mechanotransduction within the inner ear.

### Monoallelic loss of radixin increases pre-pulse inhibition

**FIGURE 6:**
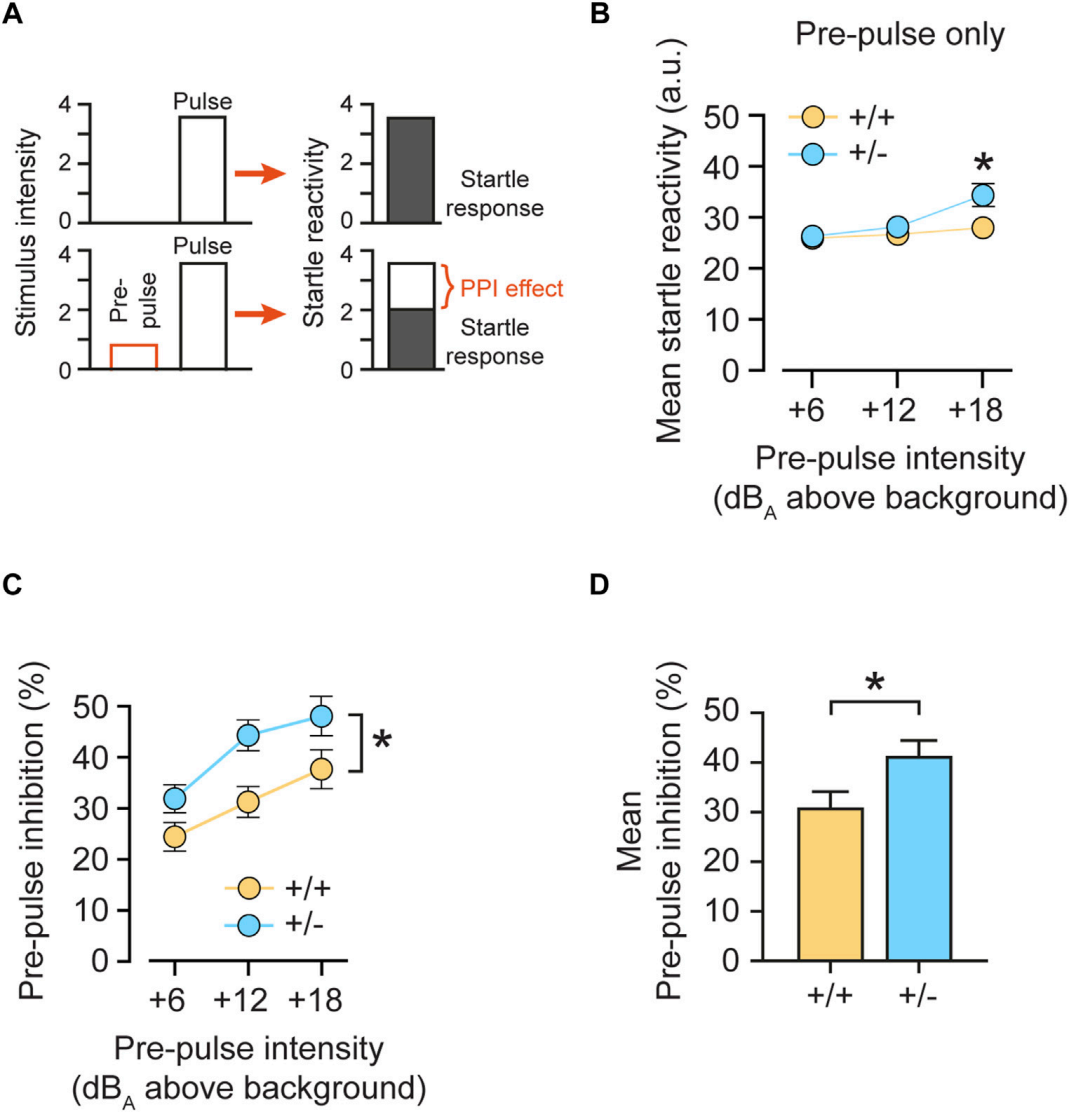
Monoallelic loss of rdx increases pre-pulse inhibition. **(A)** Schematic presentation of the pre-pulse inhibition (PPI) effect. A low pre-pulse (stimulus intensity) reduces the response to the subsequent startle-eliciting pulse. **(B)** Mean startle reactivity obtained on pre-pulse-alone trials as a function of pre-pulse intensity (dB_A_ above background noise level of 65 dB_A_). **(C)** Pre-pulse inhibition (PPI) expressed as percent inhibition (percent startle reduction relative to pulse-alone trials) plotted as a function of pre-pulse intensities (dB_A_ above the background noise level of 65 dB_A_). **(D)** Mean PPI as percent of inhibition across pre-pulse intensities illustrated in **(C)**. Heterozygous (+/-) *rdx* knockout mice are characterized by higher pre-pulse inhibition, compared to wildtype (+/+) littermate controls. Data were obtained from *n* = 16 (+/+), 16 (+/-) and 16 (−/−) adult *rdx* knockout animals. Equal numbers of males and females were used for each genotype. Repeated measurement ANOVA was used to assess statistical significance. *p < 0.05. Data represent mean ± SEM.

We therefore investigated central auditory signal integration by analyzing sensorimotor gating and tested pre-pulse inhibition (PPI) in mice. PPI is a phenomenon in which a weaker pre-stimulus (pre-pulse) inhibits the animal´s startle response to a subsequent intense startle-eliciting pulse stimulus administered ∼100 ms later (Figure 6A). PPI is regulated by the limbic cortices, the ventral striatum, the ventral pallidum, and the pontine tegmentum, and its deficiency has been linked to poor sensorimotor gating (Swerdlow et al., 2001). PPI (operationally defined as a suppression of the startle response to the pulse stimulus) reflects a top-down process triggered by a weak pre-pulse to gate, or filter out, the processing of the succeeding pulse stimulus, known as sensorimotor gating. Here, feedforward inhibition, in which a pre-pulse activates the cochlear root and the nucleus reticularis pontis caudalis (PnC), is one of the most widely accepted neural mechanisms of PPI (Koch et al., 1993).

To test whether monoallelic loss of *rdx* (+/-) could enhance sensorimotor gating, we analyzed PPI expression as indexed by the percentage of inhibition of the startle reactivity (Figure 6A). To obtain a measure of pre-pulse-elicited reactivity, we first analyzed the data obtained in pre-pulse-alone trials (Figure 6B). A 3-way ANOVA (genotype x sex x pre-pulse-alone intensity) revealed an interaction of genotype x pre-pulse-alone [F(2,56) = 6.203, *p* = 0.004]. A restricted analysis of the three pre-pulse-alone intensities (above background) showed a significant increase in the reactivity elicited by weak prepulses in heterozygous (+/-) animals, as compared with control (+/+) mice at +18 dB(A) (*p* < 0.05), but not at +6 dB(A) (*p* = 0.804) and +12 dB(A) (*p* = 0.337). In a subsequent analysis of the PPI effect, increasing intensities of the pre-pulse stimulus predicably induced stronger PPI in both genotypes (Figure 6C). However, heterozygous (+/-) animals showed a significant increase in %PPI as compared with control (+/+) mice over all three pre-pulse intensities. These results were confirmed by a 2 × 2 × 3 x 3 (genotype x sex x pulse intensity x pre-pulse intensity) 4-way ANOVA, which revealed a significant effect of pre-pulse intensities [F(2,112) = 55.815, *p* < 0.0001] and of genotype [F(1,28) = 5.803, *p* < 0.05]. Since the increase in response observed in pre-pulse-alone trials (Figure 6B) was only visible at the strongest pre-pulse (+18 dB(A)), we inferred that it could not solely explain the observed potentiation of PPI expression seen regardless of pre-pulse intensities (Figure 6C). Furthermore, an analysis of mean %PPI over three pre-pulse intensities showed a significant increase in (+/-) mice, as compared to (+/+) littermates (Figure 6D; *p* = 0.023). Finally, we detected a main effect of sex [F(1,28) = 5,702, *p* < 0.05] but no interaction with genotype [F(2,28) = 1.100, *p* > 0.3]. Therefore, our data point to increased sensorimotor gating in heterozygous *rdx* knockout mice.

### Monoallelic loss of radixin reduces its protein expression levels in PPI relevant brain regions

**FIGURE 7:**
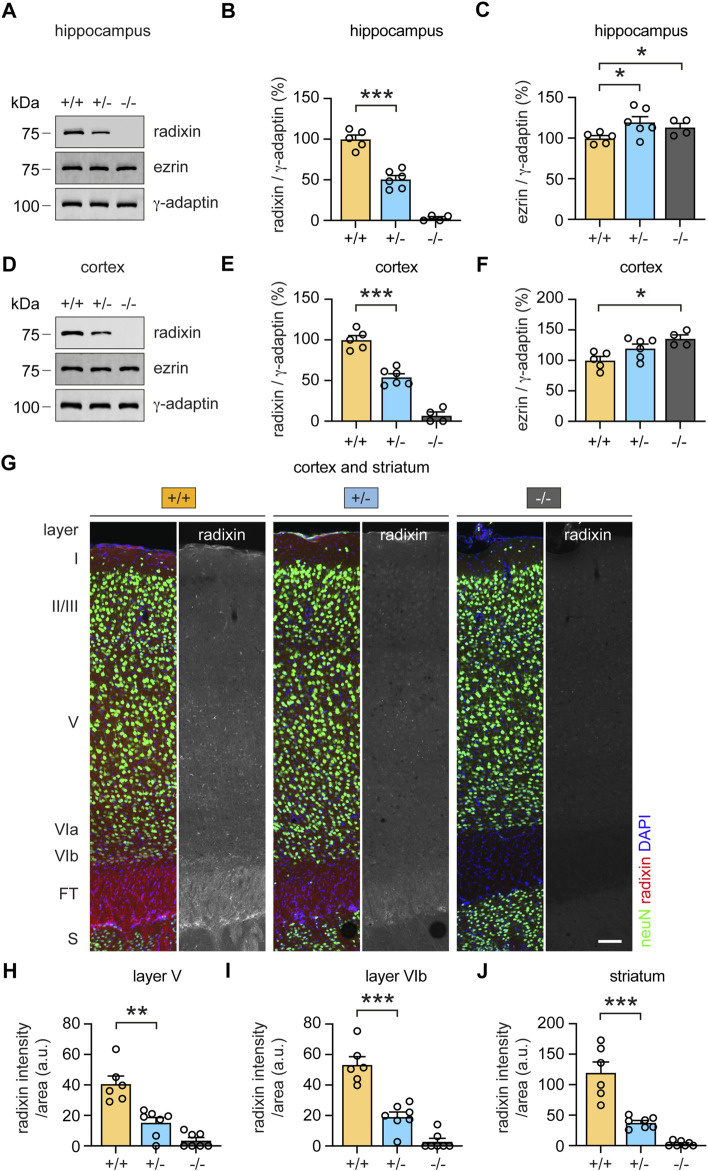
Monoallelic loss of rdx reduces radixin protein expression in the cortex, hippocampus and striatum. **(A)** Representative western blot analysis of radixin (Rdx) and ezrin (Ezr) in hippocampal extracts derived from adult (+/+), (+/-) and (−/−) *rdx* knockout mice. *γ*-adaptin was used as loading control. Kilodalton (kDa). **(B,C)** Quantification of Rdx **(B)** and Ezr **(C)** signal intensities normalized to *γ*-adaptin as shown in **(A)**. (+/+) set to 100%. N = 4-6 mice per genotype. **(D)** Representative western blot analysis of Rdx and Ezr in cortical extracts derived from adult (+/+), (+/-) and (−/−) *rdx* knockout mice. *γ*-adaptin was used as loading control. Kilodalton (kDa). **(E,F)** Quantification of Rdx **(E)** and Ezr **(F)** signal intensities normalized to *γ*-adaptin as shown in **(D)**. (+/+) set to 100%. N = 4-6 mice per genotype. **(G)** Immunohistochemical analysis of Rdx (red or gray) protein expression levels in the frontal cortex and the striatum in sagittal brain sections derived from adult (+/+), (+/-) and (−/−) *rdx* knockout mice. Layers and regions were identified based on neuN (green) and DAPI (blue). Scale bar, 100 µm. **(H–J)** Quantification of Rdx signal intensities normalized to the area analyzed in cortical layer V **(H)**, cortical layer VIb **(I)** and the dorsal cell layer of the striatum **(J)**, as shown in **(G)**. Fiber tracts (FT), striatum (S). 6-7 sections derived from 3 mice per genotype were analyzed. Independent Student´s *t*-test was used to asses statistical significance. *p < 0.05, **p < 0.01, ***p < 0.001. Data represent mean ± SEM.

To examine a putative role of radixin in central brain regions, we first analyzed Rdx protein expression levels in the hippocampus and frontal cortex. Western blot analysis confirmed the presence of Rdx in both, hippocampal and frontal cortex derived lysates of wild-type (+/+) mice, reduced expression in heterozygous (+/-), and no expression in knockout (−/−) derived lysates (Figures 7A,B; *p* < 0.0001; Figures 7D,E; *p* < 0.0001). The analysis of Ezr revealed a slight but statistically significant upregulation of protein expression levels in (+/-) and (−/−) mice in comparison with (+/+) mice in both, hippocampal and cortical derived lysates, suggesting a compensatory effect (Figures 7A,C; *p* = 0.039 (+/-); *p* = 0.047 (−/−)); Figures 7D,F; *p* = 0.066 (+/-); *p* = 0.0055 (−/−)). We further investigated a region-specific expression of Rdx within the frontal cortex and the striatum, two areas that contribute to the modulation of sensorimotor gating (Yee, 2000; Swerdlow et al., 2001; Pothuizen et al., 2006). To this end, we analyzed Rdx protein expression levels in immuno-stained sagittal brain sections. The neuron-specific marker protein neuN was used to identify individual frontal cortical layers (characterized by the absence of layer IV) and to distinguish neurons from glial cells (Mullen et al., 1992), whereas DAPI was applied to visualize cell nuclei. Confocal microscopy revealed Rdx-specific signal intensities throughout the cortex, in the fiber tracts and the striatum of wild-type (+/+) mice (Figure 7G, left). Heterozygous (+/-) mice, displayed reduced radixin signal intensities (Figure 7G, middle), while the protein was undetectable in homozygous (−/−) knockout mice (Figure 7G, right). Notably, radixin expression was enriched in cortical layer V, layer VIb and the fiber tracts, and was even more prominent in the dorsal striatal glia cells (neuN-negative). Consistent with the western blot analysis (Figures 7D,E), quantification of cortical and striatal Rdx signal intensities were reduced by about 50–60% in *rdx* (+/-) mice relative to wildtype (+/+) control animals (Figure 7H; *p* = 0.0012; Figure 7I; *p* < 0.0001; Figure 7J; *p* = 0.0004). Cortical layers V and VIb as well as the striatum regulate PPI (Kodsi and Swerdlow, 1995; Swerdlow et al., 2001; Kim et al., 2021). Therefore, it is reasonable to speculate that the reduced expression of Rdx expression levels in these regions may contribute to the startle reactivity and sensorimotor gating phenotypes in the *rdx* (+/-) mice. However, further experiments are required to specify the cellular functions of Rdx in these processes.

In summary, we showed that a monoallelic loss of *rdx* potentiated startle reactivity (Figure 2) and PPI (Figure 6), and that these phenotypes were not explicable by the observed alterations in sensory hearing transduction (Figures 4, 5). It is therefore likely that reduced Rdx levels in PPI-critical brain regions (Figure 7) could interfere with top-down auditory signal processing and/or sensorimotor integration.

## Discussion

This study proposes a role of Rdx in central auditory signal processing in addition to its well-known functions in stereociliar development, structural integrity and acoustic stimuli transduction. As shown previously, a homozygous *rdx* knockout in mice leads to the total degeneration of stereocilia within 40-days after birth, resulting in complete deafness (Kitajiri et al., 2004). Interestingly, despite about 50% protein expression levels in the cochlea, heterozygous *rdx* knockouts did not display an intermediate hearing phenotype. Instead, we observed a gain-of-function effect regarding startle reactivity, triggered by acoustic stimuli as well as its suppression by weak acoustic pre-pulse stimuli. This result is consistent with a non-linear gene/protein-dosage effect, similar as observed for other individual genes (Burgis and Gessner, 2007; Welch et al., 2020). Although we revealed a mild increase in stereocilia width, rdx (+/-) mice only showed minimal hearing impairment (Figure 5). We therefore conclude that 50% *rdx* gene dosage and protein expression levels are in general sufficient for cochlea development and stimuli transduction. On the other hand, a recent study reported an impairment of outer hair cell stereocilia function in guinea pigs, following chemical inhibition of Rdx (Prasad et al., 2020). In this study, the authors specifically disrupted radixin’s ability to link F-actin with the plasma membrane, leading to decreased sound-evoked electrical potentials. They therefore suggested a critical function for Rdx in inner ear stereocilia stiffness and mechanoelectrical transduction to convert sound to electrical signals. The stronger effects reported by Prasad and colleagues differ from our observations, which may be readily explained by the different individual approaches. First, acute pharmacological manipulation might exert fundamentally different effects, as compared to a long-term constitutive reduction of Rdx, where developmental compensation might mitigate the phenotype. For example, although expressed at very low levels in the cochlea (Kitajiri et al., 2004; Kahsai et al., 2010), one cannot exclude compensation by the Rdx-homologues ezrin and moesin in heterozygous (+/-) *rdx* knockouts. Indeed, we observed a slight, but significant increase of ezrin expression levels, that was nonetheless insufficient to rescue the deafness in *rdx* (−/−) mice. Second, species differences (*Cavia porcellus* vs *Mus musculus*) might contribute to the differing observations. Finally, chemical Rdx blockade by Prasad and colleagues might have inhibited large amounts of cochlear Rdx, thereby reducing its functional levels to less than 50%. It is therefore possible that normal stereociliar development and cochlear function requires a narrow range of radixin gene expression.

Based on the morphological and electrophysiological characterization of (+/-) *rdx* stereocilia, we have excluded major alterations in inner ear sensory mechanotransduction as a potential cause of the observed increase in acoustic startle response (ASR). Auditory brainstem response (ABR) waves correspond to the activation of the auditory nerve (wave I), which connects the cochlea to the cochlea nucleus (wave II) and further projects to the pons (superior olivary complex; wave III). Thereafter signals are transferred *via* the lateral lemniscus (wave IV), to the midbrain and inferior colliculus (wave V) and finally to the auditory cortex. Startle reflexes are elicited by the direct activation of spinal cord motor neurons through bilateral projections *via* the trapezoid body (in the medulla oblongata) to the neurons in the pontine reticular nucleus (in the pons), representing the main auditory tract that connects the super olivary complex with the inferior colliculus (Moller, 1994; Chen et al., 2010; Sekiya et al., 2015; Gomez-Nieto et al., 2020). The increase in ABR thresholds and the reduction of wave I amplitudes cannot explain the increase in startle reactivity observed with heterozygous (+/-) *rdx* mutants. Instead, our data would have predicted a decrease in startle response, which was not observed.

Since the peripheral encoding of acoustic stimuli turned out to be mainly normal, we further asked whether auditory signal gating/filtering might be altered in heterozygous *rdx* knockout animals. The startle reflex is associated with several forms of behavioral plasticity, including sensitization, habituation, conditioning, and pre-pulse inhibition (PPI) (Yeomans and Frankland, 1995; Koch, 1999). PPI refers to the inhibition of ASRs to a startle-eliciting acoustic pulse stimulus (100 dB and above here), due to a preceding weak and typically non-startling pre-pulse stimulus. This reduction in ASR provides an operational measurement of sensorimotor gating (Swerdlow et al., 2001; Gomez-Nieto et al., 2020). Interestingly, PPI was potentiated in heterozygous (+/-) *rdx* knockout mice, indicating stronger (or more sensitive) sensorimotor gating. In addition to the above-mentioned neuronal circuits involved in ASR regulation, PPI modulation includes several other brain areas such as the nucleus accumbens, the ventral pallidum, the basolateral amygdala, the mediodorsal thalamus, the medial prefrontal cortex and the hippocampus. Moreover, neurotransmitters and neurotransmitter receptors (e.g., dopamine and NMDA receptors), neuropeptides and regulatory proteins, that are widely distributed between pons and frontal cortices, modulate the expression of PPI (Caine et al., 1992; Swerdlow et al., 2001).

Since one cannot explain the observed phenotype of heterozygous (+/-) *rdx* mutants in terms of enhanced sound transduction as such, the PPI phenotype may suggests an alteration in the filtering of auditory signals and/or central gating mechanisms. Indeed, in addition to its expression in the cochlea, Rdx is abundantly expressed in many brain areas, including the pons, the medulla oblongata, the spinal cord and the cortex (Moon et al., 2013). We confirmed and extended these observations, demonstrating protein expression of Rdx in the frontal cortex and striatum. Notably, radixin expression was very prominent in the dorsal striatal glia cells. It has been shown that glial cells perform important functions regulating striatal dopamine output (Tome et al., 2007; Roberts et al., 2022), one of the most important neurotransmitters involved in PPI modulation (Kodsi and Swerdlow, 1995; Swerdlow et al., 2001). Hence, although the exact mechanisms and pathways remain to be investigated, our data indicate a significant role of Rdx in the regulation of PPI expression, possibly at multiple loci within the circuits underlying PPI of the acoustic startle reflex.

As we and others have shown, Rdx is also highly expressed in the hippocampus (Paglini et al., 1998; Kawaguchi et al., 2017), a brain region known to control the expression of PPI (Caine et al., 1992; Swerdlow et al., 1995; Pouzet et al., 1999). The molecular mechanisms by which differential ERM protein levels impact on neuronal function are largely unknown and likely diverse. For instance, ERM proteins may modulate glia-synaptic interactions by regulating the formation of peripheral astrocyte processes as well as glial glutamate uptake or dopamine release (Derouiche and Geiger, 2019; Roberts et al., 2022). Dendritic filopodia motility during neuronal synapse formation may also be influenced by ERM function (Furutani et al., 2007; Furutani et al., 2012). We previously identified Rdx as a scaffold protein for alpha5-containing gamma-aminobutyric acid (GABA)_A_ receptors (GABA_A_Rs) and demonstrated that Rdx is essential for the localization of these receptors at extrasynaptic plasma membrane sites, underlying the maintenance of tonic GABAergic inhibition (Loebrich et al., 2006). Furthermore, the inactivation of Rdx releases extrasynaptic alpha5-containing GABA_A_Rs from F-actin anchoring that leads to their relocation into synaptic sites, thereby potentiating inhibitory GABA_A_R postsynaptic currents (Hausrat et al., 2015). A recent study further reported that Rdx-mediated re-localization of alpha5-containing GABA_A_Rs acts as a mechanism to prevent over-excitation during the formation of excitatory long-term potentiation (Davenport et al., 2021). GABA_A_Rs are highly expressed in multiple regions of the auditory pathway (Campos et al., 2001), known to be involved in the modulation of PPI. Accordingly, knockout studies revealed a functional role of GABAergic innervation in the cochlea *via* alpha5-, beta2-and beta3-, but not for alpha1-, alpha2-, alpha6-and delta-containing GABA_A_ receptors (Maison et al., 2006). It is further of relevance that an intra-hippocampal infusion of the GABA_A_R antagonist picrotoxin attenuates PPI, pointing to an involvement of hippocampal GABA_A_R activity in the modulation of PPI expression (Bast et al., 2001). Notably, the highest GABA_A_R-alpha5 expression levels (encoded by *gabra5*) are reported in the hippocampus (Sur et al., 1998; Sur et al., 1999); and *gabra5* was identified as the most abundantly expressed GABA_A_ receptor gene in the superior olivary complex (Fischer et al., 2019). Hence, this GABA_A_R subunit is strategically located to influence PPI in relevant brain regions. Finally, a direct involvement of alpha5-containing GABA_A_Rs in the modulation of PPI has been demonstrated in alpha5(H105R) mutant mice. The specific loss of hippocampal alpha-5 subunit-containing GABA_A_ receptors in these mutants was associated with deficient PPI (Hauser et al., 2005). Reduced expression of Rdx in heterozygous (+/-) *rdx* knockout mice (characterized by potentiated PPI; our study, see Figure 6) is expected to release more alpha5-containing GABA_A_ receptors into synaptic sites and, should result in opposite effects on PPI as compared to the alpha5(H105R) mutant mice (characterized by reduced levels of alpha5-containing GABA_A_ receptors), which was indeed the case. Rdx might therefore control the distribution and transmission of alpha5-containing GABA_A_Rs receptors in relevant brain regions to regulate PPI. However, additional experiments are required to further dissect these region-specific roles of Rdx in regulating the expression of alpha5-containing GABA_A_Rs receptors and consequently PPI.

Notably, disruption of PPI has been demonstrated in several psychiatric diseases, including obsessive compulsive disorders, schizophrenia and autism (Braff et al., 2001; Geyer et al., 2001; Cheng et al., 2018), characterized by an overload of sensory information (Perry and Braff, 1994). Whereas intact peripheral hearing is required to encode sound, various central pathways contribute to higher-level processing of different aspects of auditory perception. They include sound localization, intensity, frequency, amplitude modulations and finally sound awareness, involving top-down and feedforward mechanisms. In summary, our combined data suggest a hitherto unknown role of Rdx in central sensory processing and/or filtering as well as gating of sound, providing a stepping stone to further investigate Rdx-mediated mechanisms in central auditory function under normal and disease conditions.

## Data Availability

The raw data supporting the conclusion of this article will be made available by the authors on request, without undue reservation.
